# Incidental mood state before dissonance induction affects attitude change

**DOI:** 10.1371/journal.pone.0180531

**Published:** 2017-07-14

**Authors:** Marie-Amélie Martinie, Yves Almecija, Christine Ros, Sandrine Gil

**Affiliations:** Université de Poitiers, Université de Tours, Centre National de la Recherche Scientifique, Centre de Recherches sur la Cognition et l’Apprentissage (CeRCA - CNRS UMR 7295), Poiters, France; Leiden University, NETHERLANDS

## Abstract

The way that incidental affect impacts attitude change brought about by controlled processes has so far been examined when the incidental affective state is generated after dissonance state induction. We therefore investigated attitude change when the incidental mood occurs prior to dissonance state induction. We expected a negative mood to induce systematic processing, and a positive mood to induce heuristic processing. Given that both systematic processing and attitude change are cognitively costly, we expected participants who experienced the dissonance state in a negative mood to have insufficient resources to allocate to attitude change. In our experiment, after mood induction (negative, neutral or positive), participants were divided into low-dissonance and high-dissonance groups. They then wrote a counterattitudinal essay. Analysis of their attitudes towards the essay topic indicated that attitude change did not occur in the negative incidental mood condition. Moreover, written productivity–one indicator of cognitive resource allocation–varied according to the type of incidental mood, and only predicted attitude change in the high-dissonance group. Our results suggest that incidental mood before dissonance induction influences the style of information processing and, by so doing, affects the extent of attitude change.

## Introduction

In daily life, we try to keep our attitudes and behaviours in harmony, as we are motivated to be consistent [1, 2, 3]. Cognitive dissonance theory [4] concerns the effect of cognitive inconsistency on attitude and behaviour. Cognitions represent individuals’ beliefs, opinions and knowledge about their behaviours, selves or environment. Inconsistencies emerge when individuals behave in a way that is at odds with their attitudes. Awareness of inconsistency elicited in an unconstrained context [5, 6] generates a negative affective state [7, 8] labelled *dissonance state*. This negative affective state constitutes a drive state [9, 10] that individuals are motivated to reduce, one way being to modify the cognition that is the least resistant to change, namely an attitude or behaviour. The dissonance process takes place in four stages: inconsistency between cognitions (Stage 1); dissonance state (Stage 2); reduction strategy (Stage 3); and dissonance alleviation (Stage 4) [11]. Some individual factors can reduce sensitivity to dissonance states [12, 13, 14]. The goal of our research was to determine whether the nature of an incidental mood state prior to dissonance state induction affects its reduction. To our knowledge, this issue had previously only been examined for incidental moods occurring after dissonance state induction.

A dissonance situation is a decision situation in which individuals freely choose to produce behaviour that is inconsistent with their initial attitudes or motivations. For instance, individuals decide to stop smoking. The dissonance process has mainly been examined in the compliance paradigm [15], in which participants are active agents of the cognitive inconsistency. Participants produce an essay that runs counter to their pre-attitude (i.e., counterattitudinal essay) (Stage 1). They have the choice to accept or refuse to realize it (i.e., high-dissonance condition) or they have to realize it (i.e., low-dissonance condition). Deciding to produce discrepant behaviour triggers undifferentiated arousal [16]. If it is labelled negatively, it becomes a dissonance state (Stage 2). The affective component of the dissonance state has been measured by self-report [7], brain imaging [17], and facial EMG [8]. For instance, our team recorded participants’ facial EMG during the completion of a counterattitudinal essay in high- and low-dissonance conditions [8]. It was observed an increase in facial muscle activity related to negative affect, but only in the high-dissonance condition.

Classically in the compliance paradigm, participants have the possibility to justify or rationalize their discrepant behaviour to reduce their dissonance state (Stage 3). One possibility is attitude change. Participants in a high-dissonance condition have been found to change their attitude more, to make it more consistent with their essay, than those in a low-dissonance condition [18]. Attitude change is positively correlated with the intensity of negative affect [19], and after attitude change participants report a less negative affect [7]. Attitude change allows individuals to reduce their negative state (Stage 4). It can occur without the completion of the counterattitudinal essay [20, 21, 22, 23]. In sum, merely agreeing to compose a counterattitudinal essay is sufficient to observe attitude change. Attitude change does not, however, occur immediately after the decision [7], but sometime between the decision and the post-attitude measurement, for instance 1 minute after the decision [22]. The negative affect related to the dissonance state may emerge during the completion of the counterattitudinal essay [8].

Previous studies investigating the effect of incidental affect on dissonance reduction manipulated participants’ affective state *after* induction of the dissonance state, and reported findings consistent with a mood-congruency effect. This effect can be attributed to the fact that sensitivity to stimuli is enhanced when their valence matches the observer’s mood [24, 25]. According to the congruency effect, negative affect associated with a dissonance state becomes even more negative in individuals experiencing a negative incidental mood. In turn, a negative mood increases the motivation to regulate the negative affect and thus to reduce dissonance. Conversely, a positive mood neutralizes the negative affect inherent to dissonance, resulting in less motivation to regulate the negative affect and thus to reduce the dissonance state.

Several studies support these predictions. For instance, some authors [26] noticed that participants exposed to a humorous cartoon after freely completing counterattitudinal essay did not change their attitude. Other authors [27] also observed that post-decisional dissonance combined with mood affects the need for dissonance reduction. In their study, all participants had to make a choice between attractive alternatives (difficult choice). A positive or negative mood was then induced by a video. In the post-manipulation phase, participants could justify their choice because they could seek information related to their choice. Results showed that negative mood combined with post-decisional dissonance increased the need to seek information, while positive mood decreased it. To sum up, evidence suggests that incidental mood influences the motivation for affect regulation: with negative mood, participants are more motivated to reduce the negative affect, whereas a positive mood motivates them to maintain their affective state or neutralizes a negative threat. These results are not surprising, given that the mood was induced immediately after the motivation to reduce the dissonance state, and thus could more easily affect it.

In real life, people are already in a particular—and potentially longlasting—mood state when they encounter dissonant situations. In contrast with previous studies, we therefore asked what happens when an incidental mood state occurs before—and not after—the induction of a dissonance state. According to the *feelings-as-information hypothesis* [28], affective states tell people about their current environment. Negative moods indicate that the environment is problematic or threatening. Individuals try to eliminate the threat of negative outcomes, using a detail-oriented information processing style that involves selective attention. If people are in a positive mood, they do not need to use this particular processing style, as their positive mood tells them that the environment is safe and satisfactory. They therefore use simple heuristics and have a broader attentional scope.

One major consequence of this hypothesis is that the valence of the affective state determines the nature of the information processing style. Negative mood elicits systematic processing, whereas positive mood elicits heuristic processing [29] Systematic processing involves meticulously and rationally taking all the information into consideration, and is therefore cognitively costly [30]. By contrast, heuristic processing involves the use of more superficial styles of thinking, thus allowing individuals to save their cognitive resources for other tasks. For instance, in the case of persuasion, where individuals are passive receivers, participants who are in a negative mood change their attitudes in response to strong arguments but not weak ones, whereas those who are in a positive mood change their attitudes whatever the strength of the arguments [31]. When attitude change varies according to argument strength, it suggests that the individuals are engaged in systematic message elaboration [30]. Moreover, according to the feelings-as-information hypothesis, when people are aware that their mood can be attributed to an irrelevant source, these effects cease to be observed [32]. The misattribution of mood to an irrelevant source argues in favour of a relationship between emotional experience and social information processing.

In the framework of cognitive dissonance, the dissonance state (Stage 2) has been shown to have energizing properties [9, 10], and so consumes few resources itself [19]. For instance, our team realized a study in which participants produced a counterattitudinal essay in a high- or low-dissonance condition [19]. As they did so, they performed a memory-load task in which they had to retain either three (low-load) or five (high-load) items. Results showed that under a low-load condition, performances on the memory task were just as good in the high-dissonance condition as in the low-dissonance one. By contrast, under a high-load condition, performances were poorer in the high-dissonance condition than in the low-dissonance one. In sum, writing a counterattitudinal essay in a high-dissonance condition only slightly reduces working memory capacity, and therefore consumes few resources per se.

According to cognitive dissonance theory [4], attitude change (Stage 3) involves consciously controlled processing, as individuals need to make a deliberate effort to justify their counterattitudinal behaviour. Results support this perspective. For instance, attitude change ceases to be observed if the participant does not focus on the dissonant elements [33]. Moreover, dissonance affects explicit, but not implicit, attitudes [34]. *Explicit* attitudes involve cognitively costly control processes, whereas *implicit* attitudes involve less costly automatic processes. Finally, the brain mechanisms related to conflict reduction (which have been localised in the anterior cingulate cortex), do not occur immediately after individuals agree to produce inconsistent behaviour and even while they are performing it, but they occur when participants report their post-attitudes [17]. These results are consistent with those obtained by some authors [7, 22]. They observed no attitude change when the post-attitude measurement appeared just after the decision to realize the counter-attitudinal essay [7]. However, attitude change occurs when one minute is introduced between the decision and the post-attitude measurement [22]. Taken together, these results suggest that, in the compliance paradigm, the mechanism leading to attitude change is based on controlled processes, not automatic ones.

As mood dictates the information processing style (i.e., systematic for negative mood vs. heuristic for positive mood), we would expect it to affect attitude change, which is based on controlled processes [17, 33, 34]. More specifically, in a high-dissonance condition, because of the low cognitive cost of producing a counterattitudinal essay [19], individuals in a negative mood can afford to engage in systematic information processing—which is cognitively costly—before reporting their post-attitudes. Because it is based on controlled processes, attitude change consumes resources, so given that people have finite cognitive resources [35], we would expect a negative mood (which induces systematic processing) to affect attitude change. To test this hypothesis, we measured participants’ pre-attitudes towards the essay topic. We then randomly assigned them to one of three mood induction conditions (positive, neutral or negative). Within each condition, participants were divided into a low-dissonance group and a high-dissonance one, in which they were given 5 minutes to produce a counterattitudinal essay. We then measured their attitude towards the essay topic again. Because a negative mood elicits systematic processing, which involves analysing all the information pertaining to the situation, unlike heuristic processing (corresponding to a positive emotional state), we would expect it to lead individuals to elaborate their thinking by considering both sides of the question (arguments for and against). Consequently, insofar as participants only had to argue one side (arguments for) of the question in their essay, this unilateral written production would only partially reflect this elaboration processthinking. Fewer resources would therefore be allocated to the unilateral written production in the negative mood condition. To check the type of information processing that participants performed before reporting their post-attitude, we considered resource allocation by examining an indicator of investment in written production: syntactic complexity [36]. The less syntactically complex the essay, the fewer resources participants would have allocated to its production [37]. And the less complex the essay, the more participants would have engaged in systematic processing before reporting their post-attitude. Consequently, we expected to observe a main effect of induced mood, with low complexity for negative mood, average for neutral mood, and high for positive mood. Crucially, for attitude change, we expected to observe an interaction between group and mood. We predicted that in the high-dissonance group, participants in a negative mood would maintain their initial attitude contrary to those in a neutral or positive mood. Moreover, the type of information processing would only predict attitude change in the high-dissonance group.

## Method

### Participants

We conducted the present study in accordance with the principles expressed in the Declaration of Helsinki. Written consent was obtained from all the participants. Our study was approved by the Tours-Poitiers ethics committee for noninterventional research (no. 2016-09-01). We recruited 132 psychology students in exchange for course credits. Participants (*n* = 16) who did not follow the instructions were excluded from the analyses: three participants failed to return to the laboratory for the second phase; eight refused to write the essay; and five wrote a pro-attitudinal essay. The final sample therefore comprised 116 participants (i.e., high-dissonance group: 18 with negative mood, 24 with neutral mood, 17 with positive mood; low-dissonance group: 21 with negative mood, 17 with neutral mood, 19 with positive mood).

### Materials

We created six movie clips, two for each type of induced mood (i.e., negative, positive, neutral), based on a validated set of films [38] and the videos recommended for neutral mood induction [39]. As usual with mood induction via movies, each clip lasted about 10 minutes [40].

We administered the validated French version [41] of the Brief Mood Introspection Scale (BMIS) [42], which allows two emotional dimensions to be assessed: pleasant-unpleasant and arousal-calm. Participants indicated how far each item described their present mood on a scale ranging from 1 (*Definitely do not feel it*) to 4 (*Definitely feel it*).

### Design and procedure

To avoid making the pre-attitude salient and thus resistant to change [43], the participants’ pre-attitude towards a 4-year Bachelor’s degree course was measured on a scale ranging from 0 (*Totally against*) to 9 (*Totally in favour*) 7 days before the experimental phase. This measure revealed that participants were opposed to a 4-year course (*M* = 1.89, *SD* = .94). In the experimental phase, participants first completed the BMIS (i.e., baseline mood). They then viewed the first of two movie clips (i.e., one clip from one of the pairs created for the three mood induction conditions), having been instructed to watch the clip quietly and carefully because they would have to answer some questions afterwards. To introduce the clip, they were told that the purpose of the study was to examine auditory and visual memory. Thereafter, they completed the BMIS again (i.e., mood assessment). They then viewed the second clip, after which participants composed an essay in favour of the 4-year Bachelor’s degree. Dissonance was induced by giving participants the choice to accept or refuse to write the essay, in accordance with classic studies of dissonance. The experimenter said: “We have found that one of the best ways of finding out the arguments on either side of a controversial issue is to have people write strong and forceful essays putting forward one side of the question.” In the high-dissonance condition, the experimenter said, “We already have enough people arguing against the 4-year Bachelor’s degree, so what we would like you to do, if you are willing, is to write an essay arguing that the 4-year Bachelor’s degree is a good idea for students. Are you willing to do that now?” The low-dissonance condition was identical to the high-dissonance condition, except that the experimenter omitted the clause “if you are willing” and did not ask participants whether they were willing to write the essay. Participants had 5 minutes to write their essay, after which we measured their post-attitude. At the end of the experiment, participants were debriefed about the purpose of the experiment and, where necessary, were exposed to the positive movie clips to neutralize the negative mood induced by the negative ones.

### Manipulation checks

#### Mood induction

For the mood manipulation check, we ran 2 (group: low dissonance vs. high dissonance) x 3 (mood: positive vs. neutral vs. negative) analyses of variance on the difference scores between induced mood and baseline mood, as measured on the pleasant-unpleasant and arousal-calm dimensions of the mood scale. On the pleasant-unpleasant dimension, we only observed an effect of mood, *F*(2, 110) = 30.54, *p* < .001, *η*^2^ = .35. As expected, in the negative mood induction condition (*M* = -7.41, *SD* = 4.79), participants rated their mood as more unpleasant than those in either the neutral (*M* = -2.77, *SD* = 3.97), *F*(1, 110) = 26.34, *η*^2^ = .19, or positive (*M* = -0.41, *SD* = 3.04), *F*(1, 110) = 58.67, *η*^2^ = .34, induction conditions did. In the neutral induction condition, participants rated their mood as more unpleasant than those in the positive induction condition did, *F*(1, 110) = 7.16, *η*^2^ = .06 (all *p*s < .05).

On the arousal-calm dimension, we again only noted an effect of mood, *F*(2, 110) = 11.32, *p* < .001, *η*^2^ = .17. In the negative mood induction condition (*M* = 1.23, *SD* = 4.10), participants rated their arousal higher than those in either the neutral (*M* = -2.21, *SD* = 2.98), *F*(1, 110) = 22.60, *η*^2^ = .17, or positive (*M* = -0.36, *SD* = 2.33), *F*(1, 110) = 4.64, *η*^2^ = .04, induction conditions did. Moreover, participants in the positive mood induction condition rated their arousal higher than those in the neutral induction condition did, *F*(1, 110) = 6.20, *η*^2^ = .05 (all *p*s < .05). In sum, participants consistently reported experiencing the target mood, suggesting successful mood induction.

#### Written production

For resource allocation, we examined investment in written production, as measured by the number of clauses in each essay. A sentence contains one or more clauses. Each clause contains a verb, a subject and an object. The number of clauses was calculated using Tropes software [44, 45]. Data were first screened for outliers. One participant had a productivity score more than 3 *SD* above the mean (*z* = 3.54), and was therefore excluded from analyses (*N* = 115). The number of clauses correlated with the number of sentences (*r* = .71). A covariance analysis of the number of clauses, with number of sentences as a covariate and group and mood as predictors, revealed a predictive effect of the covariate on productivity, *F*(1, 108) = 118.45, *p* < .001, η^2^ = .52. Importantly, analysis revealed a significant effect of mood, *F*(2, 108) = 3.95, *p* < .05, η^2^ = .06, with data following a linear trend (*p* = .01). This indicated that participants in the negative mood condition had the lowest productivity (*M* = 7.15, *SD* = .40), and those in the positive mood condition had the highest (*M* = 8.55, *SD* = .52), with participants in the neutral mood condition coming in between (*M* = 7.57, *SD* = .37). We found no effect of group, and no Group x Mood interaction (all *p*s > .1). These results showed that in the negative mood condition, participants allocated fewer cognitive resources to their written production than those in either the neutral or positive conditions, suggesting that mood dictates the information processing style (negative mood: systematic processing; positive mood: heuristic processing).

## Results

**Fig 1:**
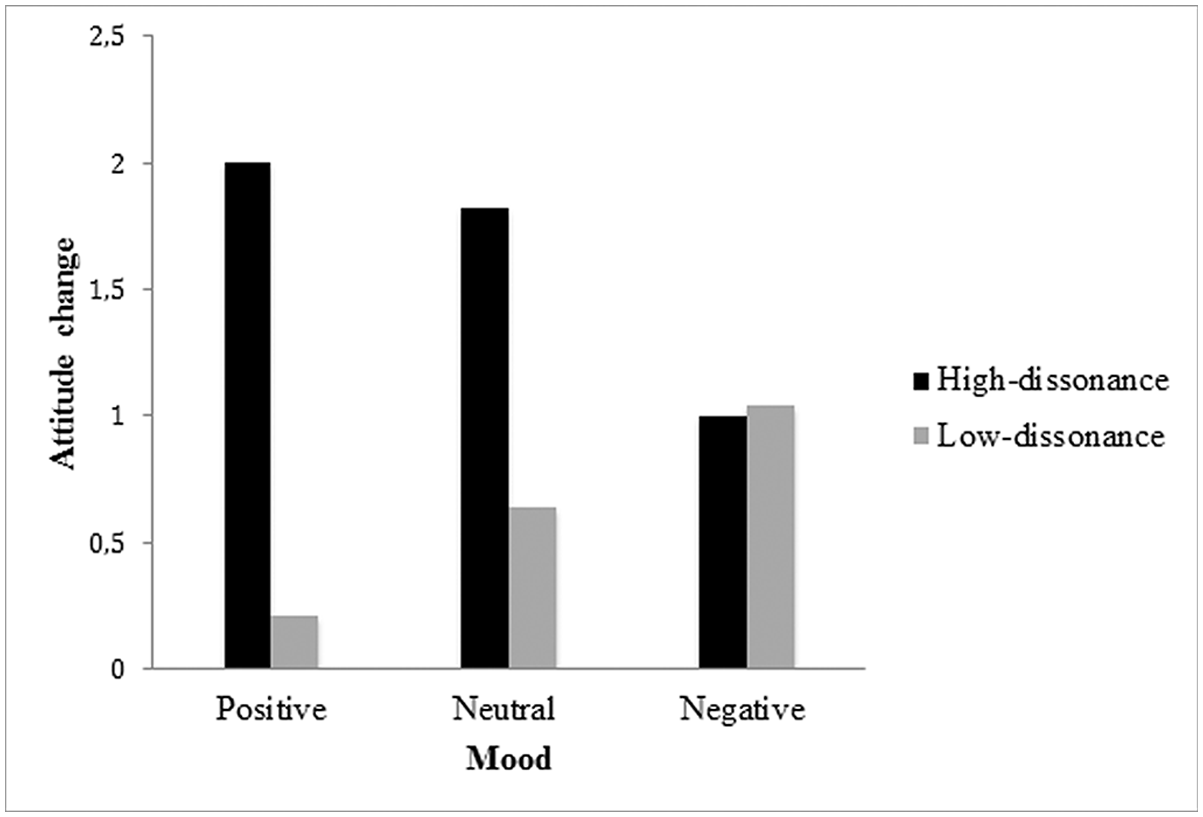
Means of attitude change for the low-dissonance vs. high dissonance group according to the valence of mood.

We ran a 2 (group: low dissonance vs. high dissonance) x 3 (mood: positive vs. neutral vs. negative) analysis of variance on attitude change (post -minus pre-attitude) that revealed a significant effect of group, *F*(1, 109) = 14.72, *p* < .001, η^2^ = .11. Participants in the high-dissonance group changed their attitude (*M* = 1.62, *SE* = .20) more than those in the low-dissonance group (*M* = 0.64, *SE* = .15). This effect showed that the manipulation of cognitive dissonance (choice whether to produce a counterattitudinal essay = high-dissonance condition; no choice = low-dissonance condition) was successful. As illustrated in Fig 1, this analysis also revealed an interaction between group and mood, *F*(2, 109) = 4.50, *p* < .01, η^2^ = .07.

In the neutral mood condition, participants in the high-dissonance group changed their attitude (*M* = 1.82, *SE* = .37) more than those in the low-dissonance group (*M* = .64, *SE* = .25), *F*(1, 109) = 7.43, *p* < .01, *η*^2^ = .06. This effect was also observed in the positive mood condition, *F*(1, 109) = 15.71, *p* < .01, *η*^2^ = .12 (high-dissonance: *M* = 2.00, *SE* = .30; low-dissonance: *M* = 0.21, *SE* = .30). By contrast, in the negative mood condition, no difference was observed between the high- (*M* = 1.00, *SE* = .32) and low-dissonance groups (*M* = 1.04, *SE* = .23), *F* < 1. Moreover, in the high-dissonance group, no difference was observed between the positive and neutral mood conditions, with similar attitude changes in both conditions, *F <* 1. However, participants in the high-dissonance group who were in a negative mood, rather than a neutral or positive one, did not change their attitude, *F*(1, 109) = 5.61, *p* < .01, *η*^2^ = .04. In sum, these results showed that, in the high-dissonance group, attitude change did not take place in the negative mood condition, in contrast to the neutral and positive ones.

**Fig 2:**
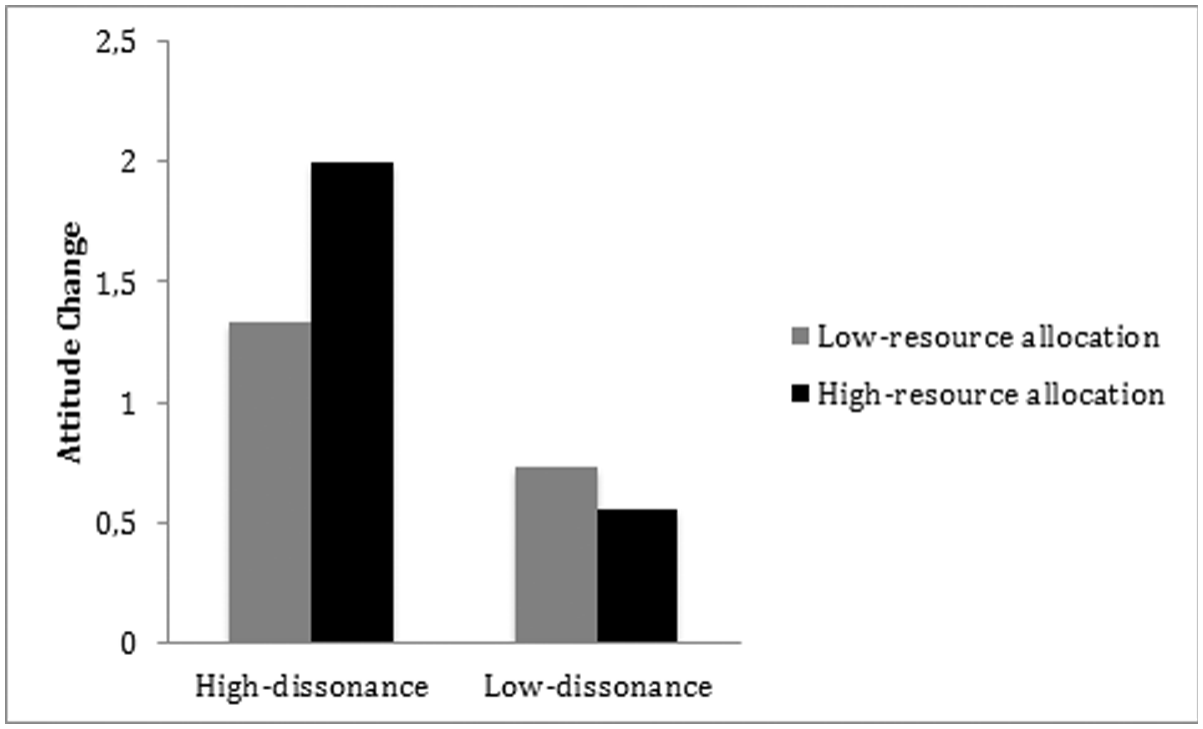
Means of attitude change for the low-dissonance vs. high dissonance group according to the level of resource allocation.

To determine whether type of information processing, as measured by the resources allocated to the counterattitudinal essay, predicted attitude change, the latter was regressed on condition (coded -1 for low dissonance and +1 for high dissonance), resource allocation to the essay, and their product term. Our results revealed a main effect of condition, *ß* = .36, *SE* = .11, *t*(114) = 3.17, *p* = .001, *d* = .59, no effect of resource allocation, *ß* = .08, *SE* = .09, *t*(114) = 0.94, *ns*, and, as expected, a significant interaction between the two, *ß* = .21, *SE* = .09, *t*(114) = 2.36, *p* = .02, *d* = .44. The data are plotted in Fig 2.

This interaction showed that, in the high-dissonance group, attitude change was greater among participants who allocated considerable resources to their essay than among those who allocated few resources. By contrast, in the low-dissonance group, resource allocation did not affect attitude change.

Correlation analyses failed to reveal any relationship between resource allocation and attitude change when the two dissonance conditions were considered together, *r* (114) = .10, *ns*. This relationship was, however, significant in the high-dissonance group, *r* (57) = .27, *p* < .05, though not in the low-dissonance one, *r* (56) = -.16, *ns*. Overall, these results indicate that type of information processing predicted attitude change. The more participants engaged in systematic processing, the less they changed their attitudes in the high-dissonance condition.

## Discussion

So far, the way in which incidental mood influences attitude change has been examined in situations where an incidental affective state occurs after dissonance state induction [26, 27]. The aim of the present study was to investigate this influence when an incidental mood occurs prior to dissonance state induction, as it does in real life. In accordance with the feelings-as-information hypothesis [28], we expected a negative mood to induce systematic processing, and a positive mood to induce heuristic processing. Given that systematic processing [30] and attitude change are both cognitively costly [34, 17] we expected a negative mood state to affect attitude change.

First, our results showed the standard effect of dissonance: attitude change was observed when participants freely produced the counterattitudinal essay, regardless of mood valence. Second, participants allocated the fewest resources to the essay in the negative mood condition, and the most resources in the positive mood condition. These results suggest that systematic processing was elicited in the negative mood condition, and heuristic processing in the positive one. Third, as expected, no attitude change was observed when participants in the high-dissonance state were in a negative mood, rather than a neutral or positive one. Fourth, when high dissonance was induced, type of information processing, as measured by the resources allocated to the essay, predicted attitude change. Accordingly, the more participants in the high-dissonance condition engaged in systematic processing, the less they changed their attitude. Taken together, our findings suggest that incidental mood influences dissonance reduction through attitude change, as individuals did not change their attitude when they were in a negative rather than a neutral or positive emotional state. As the nature of the incidental mood prior to the dissonance state determined the style of processing and, in turn, the allocation of resources, attitude change could only occur if participants had sufficient resources to allocate to it. Additional research is needed to investigate the relationship between attitude change and resource allocation.

One can postulate that in some situations, positive mood induces heuristic processing, allowing individuals to change their attitude, whereas negative mood induces systematic processing, allowing individuals to resist attitude change. This hypothesis implies that attitude change may occur automatically, if resources are not allocated to resisting it. This raises the question of the nature of the mechanisms leading to attitude change in relation to dissonance reduction. Research precisely suggests that these mechanisms are specific to the experimental situation, which may involve either automatic or controlled processes. In the framework of cognitive dissonance, attitude change is classically investigated with the compliance and free-choice paradigms. In the free-choice paradigm, participants rate several items (pre-choice preference). They are then shown pairs of items and asked to choose each time which of the two items they prefer. These pairs contain either similar (difficult choice) or dissimilar (easy choice) items. When the items are similar, the cognition “I rejected the positive features of the rejected alternative” is in a dissonant relationship with the cognition “I accepted the negative features of the chosen alternative”. The presence of these two cognitions arouses an uncomfortable psychological state (dissonance state) that participants are motivated to reduce. Finally, participants are asked to rate the stimuli a second time (post-choice preference). Individuals have been shown to change their preference after a difficult choice [5]. Attitude change has been observed after multiple choices [46], among patients with anterograde amnesia [47], and with a second cognitive task [47]. Moreover, some authors. [46] observed potential neural mechanism recruitment for conflict resolution during a difficult choice. Taken together, the results yielded by this paradigm suggest that post-choice attitude change is based on automatic processing with no deliberate effort. It can occur without any conscious recollection (i.e., explicit memory) of choices. Post-choice attitude change “is an integral part of the process leading to a choice and, as such, it occurs during the choice, not afterward” [48]. By contrast, results yielded by the compliance paradigm (the one we used) suggest that the mechanisms leading to attitude change involve controlled processes [17, 33, 34]. This argues against the hypothesis of resistance to attitude change.

Our results were not consistent with the emotional congruency effect, according to which the combination of a negative induced mood and negative affect associated with dissonance produces a high level of emotion regulation through attitude change, unlike a positive induced mood, which may neutralize the negative dissonance state. Our results do not support this congruency effect, contrary to the results obtained in previous study [49]. These authors induced an affective state prior to dissonance state induction by putting electrodes on participants’ faces. They found that when participants produced a counterattitudinal essay with electrodes on their faces to stimulate a frown (assumed to induce a negative affect), they changed their attitude more than participants in a neutral affective state did. By contrast, participants who wore electrodes on their face to stimulate a smile (i.e., positive affect) did not change their attitude. This lack of consistency between previous results [49] and our own may stem from the difference between the emotional state induction methods we used (i.e., facial feedback vs. movie clips). According to the componential model of emotion (experiential, physiological and behavioural responses), different methods may elicit a more or less intense, specific or ephemeral affect. Meta-analyses of emotion induction have shown that movie clips are one of the most effective ways of eliciting mood [50]. Additional research is needed to investigate this point by comparing different elicited incidental emotional states and their impact on the dissonance process.

Our results were also not consistent with the misattribution and distraction hypotheses. In our study, participants who had watched positive or negative clips reported higher arousal than those who had watched the neutral ones. Positive and negative clips are therefore both sources of arousal, which could explain the undifferentiated arousal of the dissonance state. According to the misattribution hypothesis, individuals attribute undifferentiated arousal to an arousing positive [51] or negative source [52], and therefore do not change their attitude. Similarly, according to the distraction hypothesis, no attitude change occurs when participants are distracted [33]. The distraction effect should therefore be observed with both positive and negative clips.

Furthermore, the unpleasantness of writing the counterattitudinal essay could have overshadowed the negative affect of the dissonance state. Participants in the negative mood condition might have been motivated to direct their cognitive resources away from the counterattitudinal essay, thus reducing the impact of the dissonance state and, by so doing, reducing the need for attitude change. However, had the unpleasantness of writing the counterattitudinal essay overshadowed the negative affect of the dissonance state, we would not have observed any difference between the high- and low-dissonance groups in the neutral mood. Our results were therefore not congruent with this hypothesis.

It could be argued that one limitation of our investigation was that arousal differed across the mood conditions. Although mood induction was successful, negative mood was linked to a higher degree of arousal than positive or neutral mood. However, two arguments can be put forward against the potential role of arousal. First, if arousal were indeed an explanatory factor for attitude change, then positive mood should have resulted in less attitude change than neutral mood, given that—like negative mood—it has been linked to a higher degree of arousal than neutral mood. Second, research has shown that instead of involving less attitude change, a high degree of arousal actually involves more attitude change. For example, if arousal is increased by caffeine [53, 54], attitude change increases. Conversely, if a person’s arousal state is lessened by alcohol [53] or by a tranquilizing drug [55], then attitude change is reduced. In sum, it seems that the crucial element in the present results was the valence of the mood per se. However, further research is needed to fully understand the respective roles of valence and arousal.

To conclude, our study suggests that inducing mood prior to dissonance induction impacts attitude change. This could be used by healthcare professionals when they advise individuals to make a decision (stop smoking, lose weight, or reduce their alcohol consumption) that runs counter to their attitudes or motivations. After making a decision, individuals seek to justify it through attitude change. This attitude change can lead individuals to change their behaviours [56]. The induction of a neutral or positive mood before making a decision to change negative health behaviour could help to promote positive health behaviour. Moreover, our findings open up a fresh avenue of research about why attitude change is not always observed.

## Supporting information

S1 File Raw data of attitude (pre, post) and written productivity for each group (low, high dissonance) and each condition (positive, neutral, negative mood). (PDF)
