# Supplementary material for: Incidental mood state before dissonance induction affects attitude change
Source: PLoS One. 2017 Jul 14;12(7):e0180531. doi: 10.1371/journal.pone.0180531 (PMC5510817; doi:10.1371/journal.pone.0180531)
Supplement: S1 File — (PDF) [file pone.0180531.s001.pdf]

Group	Condition	post-Attitude	pre-Attitude	Written productivity
Low dissonance	positive	6	3	5
Low dissonance	negative	4	4	8
Low dissonance	positive	5	3	6
Low dissonance	positive	1	3	10
High dissonance	negative	3	1	6
Low dissonance	neutral	2	1	5
High dissonance	positive	3	1	11
High dissonance	negative	2	1	2
Low dissonance	negative	2	1	6
High dissonance	neutral	2	1	11
High dissonance	positive	1	1	11
High dissonance	neutral	4	3	7
Low dissonance	neutral	2	2	7
High dissonance	neutral	5	1	8
Low dissonance	positive	3	4	7
High dissonance	positive	4	2	6
Low dissonance	positive	5	4	9
High dissonance	negative	4	3	8
Low dissonance	neutral	2	2	6
Low dissonance	negative	1	1	7
Low dissonance	negative	6	3	5
Low dissonance	neutral	4	2	6
High dissonance	negative	1	2	5
High dissonance	positive	3	1	3
Low dissonance	positive	1	2	7
High dissonance	neutral	2	2	**18**
High dissonance	negative	2	2	7
High dissonance	negative	1	1	4
Low dissonance	negative	2	1	3
High dissonance	neutral	5	1	12
High dissonance	positive	3	1	9
High dissonance	negative	4	1	10
Low dissonance	positive	4	3	13
High dissonance	neutral	3	1	9
Low dissonance	negative	3	2	6
High dissonance	negative	3	2	10
High dissonance	neutral	3	2	7
High dissonance	positive	5	3	7
Low dissonance	neutral	4	2	10
High dissonance	neutral	2	3	7
Low dissonance	positive	4	4	6
High dissonance	negative	3	1	9
High dissonance	positive	1	1	5
High dissonance	negative	1	2	13
High dissonance	negative	5	1	11
High dissonance	neutral	3	1	13
High dissonance	positive	5	2	7
High dissonance	neutral	3	3	6
High dissonance	neutral	5	2	7
Low dissonance	neutral	3	1	5
Low dissonance	positive	1	1	8
High dissonance	positive	4	3	3
Low dissonance	positive	5	3	11
High dissonance	positive	5	1	8
Low dissonance	positive	1	1	10
Low dissonance	negative	5	3	11
High dissonance	neutral	1	1	6

High dissonance	negative	3	1	7
High dissonance	neutral	6	3	7
Low dissonance	neutral	2	1	5
Low dissonance	positive	1	3	6
Low dissonance	positive	4	3	6
Low dissonance	negative	1	1	11
High dissonance	negative	3	1	4
High dissonance	negative	2	3	7
Low dissonance	negative	2	1	3
Low dissonance	negative	3	2	6
High dissonance	positive	3	3	8
Low dissonance	neutral	3	2	9
High dissonance	positive	4	3	8
Low dissonance	neutral	3	1	8
Low dissonance	negative	2	2	7
Low dissonance	positive	2	1	13
Low dissonance	positive	1	1	4
Low dissonance	neutral	2	1	8
High dissonance	negative	2	1	7
High dissonance	negative	4	3	11
Low dissonance	positive	1	1	12
Low dissonance	negative	4	4	4
Low dissonance	positive	1	2	8
Low dissonance	negative	2	2	8
High dissonance	neutral	2	3	8
Low dissonance	negative	1	1	9
High dissonance	neutral	6	3	7
Low dissonance	negative	4	3	6
High dissonance	neutral	2	1	8
Low dissonance	neutral	2	1	6
Low dissonance	negative	3	1	7
High dissonance	neutral	5	1	7
Low dissonance	neutral	1	1	4
Low dissonance	negative	6	3	10
Low dissonance	negative	6	3	8
High dissonance	positive	4	1	7
High dissonance	neutral	4	1	7
High dissonance	neutral	5	1	6
Low dissonance	negative	2	1	8
Low dissonance	neutral	1	3	13
High dissonance	neutral	7	2	11
High dissonance	neutral	3	2	6
Low dissonance	neutral	2	2	11
Low dissonance	positive	3	3	9
High dissonance	positive	3	1	15
High dissonance	positive	4	1	13
Low dissonance	negative	3	1	6
Low dissonance	positive	2	2	13
High dissonance	neutral	2	3	5
Low dissonance	negative	2	2	8
High dissonance	negative	2	1	5
High dissonance	negative	2	2	6
Low dissonance	neutral	1	1	5
High dissonance	neutral	2	1	4
Low dissonance	neutral	3	3	11
High dissonance	neutral	5	3	7
Low dissonance	neutral	3	3	8
High dissonance	positive	4	1	15

High dissonance	positive	6	2	9

number of clauses	Diff (induction-baseline) Pleas Unpleast
2	-2
4	-9
3	-3
4	-1
3	-14
2	-1
3	2
2	-12
2	-4
6	-5
5	0
2	-4
5	-10
3	-2
4	0
4	1
4	-3
5	-4
4	-3
4	-15
2	-10
3	-1
5	0
2	-1
3	-4
7	-2
5	-15
1	-4
1	-4
6	-10
3	3
4	-8
4	4
4	-5
2	-15
5	-5
5	0
3	-1
4	0
5	-1
3	-2
3	3
2	-1
7	-8
7	-15
6	-2
2	0
3	0
4	-3
3	-3
4	-2
2	2
4	-2
6	1
3	1
5	-7
3	3

2	-2
3	-4
4	0
4	-6
3	-1
8	-9
2	-8
3	-8
1	4
3	-2
3	0
4	2
5	5
4	3
2	-6
4	-9
3	4
4	-7
4	-3
6	-8
4	-3
2	-6
4	2
4	-3
4	-8
3	-8
4	-8
2	-10
4	-2
3	0
3	-14
3	4
2	-6
4	-12
4	-12
3	2
2	-2
3	-2
3	-10
7	-3
4	-15
5	1
4	-2
3	-5
5	-1
5	1
3	-9
8	-3
2	-7
5	-8
3	-5
5	-4
2	0
2	-3
4	-9
3	-1
3	1
8	5

3	2

diff (induction-baseline) ArousalCalm	Diff (induction-baseline)PosiTired
-2	-2
-7	-8
-4	-3
0	-2
5	-3
-1	-1
-2	0
6	0
0	-1
-7	-6
-1	0
-3	-3
-2	-6
-1	-1
-4	-2
2	1
-2	-1
9	4
-2	-2
2	-3
6	-1
0	0
-9	-4
-1	-1
-2	-3
0	-1
3	-6
-1	-2
1	-1
-5	-7
0	1
0	-3
-2	-1
-4	-4
1	-4
3	0
-5	-3
-2	-1
-3	-1
-1	-1
1	-1
3	2
1	0
2	-2
4	-3
-2	-2
2	0
-2	-1
-5	-4
-4	-4
-2	-2
0	0
2	0
3	2
0	1
-3	-4
2	3

1	0
-5	-4
-4	-2
1	-2
-2	-1
0	-3
12	4
4	-1
1	3
2	1
-1	-1
-5	-3
-1	1
-4	0
1	-2
-5	-8
6	5
-8	-7
-5	-4
1	-1
3	0
-1	-2
2	2
-1	-1
-5	-5
2	-1
-2	-5
1	-3
3	0
0	0
-4	-7
-3	0
0	-3
6	-3
5	-2
1	1
1	-1
-10	-6
1	-4
4	2
-3	-9
1	1
-1	-1
-2	-4
1	0
3	2
-3	-4
-2	-2
3	0
4	0
-5	-4
1	-1
-2	-1
-1	-2
-3	-5
-2	-1
0	0
-3	0

-1	1

Diff (induction-baseline) NegaRelax
0
5
7
1
7
0
-2
6
1
-2
0
0
3
1
-7
1
7
5
0
6
7
0
-5
0
0
1
7
1
2
1
-1
3
-2
0
5
3
-3
-1
-2
0
10
1
1
4
7
0
2
-1
-2
-1
0
0
2
1
0
1
0

	1
	-1
	-2
	3
	4
	3
	8
	5
	-1
	1
	0
	-3
	-2
	-3
	3
	0
	2
	-2
	-1
	2
	3
	5
	0
	0
	0
	3
	2
	4
	2
	0
	2
	-3
	1
	5
	3
	0
	2
	-5
	5
	3
	5
	0
	0
	6
	1
	1
	1
	0
	3
	4
	-1
	2
	-1
	1
	1
	0
	-1
	-3

-2
